# Triple sellar collision lesion: a unique case of pituitary adenoma, Rathke cleft cyst, and xanthogranuloma—case report and systematic review of the literature

**DOI:** 10.1007/s10014-025-00504-4

**Published:** 2025-06-06

**Authors:** Miguel A. Del Toro-Colín, Martha Tena-Suck, Alberto Santiago-Balmaseda, Citlalteptl Salinas-Lara, Germán Velázquez-Garcia, Maria de Lourdes Aguilar-Gómez, Elsa Yazmín León-Marroquín, Carlos Sánchez-Garibay, Alma Ortíz-Plata, Roger Carrillo-Meza, Noemi Gelista-Herrera, Lesly Hernández-Roque, Luis O. Soto-Rojas

**Affiliations:** 1 Laboratorio de Patogénesis Molecular, Laboratorio 4, Edificio A4, Facultad de Estudios Superiores Iztacala, Carrera Médico Cirujano, Universidad Nacional Autónoma de México, 54090 Tlalnepantla, Edomex Mexico; 2 Red MEDICI, Facultad de Estudios Superiores Iztacala, Carrera Médico Cirujano, Universidad Nacional Autónoma de México, 54090 Tlalnepantla, Edomex Mexico; 3 Departamento de Neuropatología, Instituto Nacional de Neurología y Neurocirugía “Dr. Manuel Velasco Suárez”, 14269 Ciudad de Mexico, Mexico; 4 Carrera de Médico Cirujano Facultad de Estudios Superiores Zaragoza, Universidad Nacional Autonoma de México, Iztapalapa, 09239 CDMX, México; 5 Progama de Doctorado en Ciencias Quimicobiológicas, Escuela Nacional de Ciencias Biológicas, Instituto Politécnico Nacional, Miguel Hidalgo, 11350 CDMX, México; 6 Departamento de Física Médica, Hospital de Oncología Centro Médico Nacional Siglo XXI, Instituto Mexicano del Seguro Social, CDMX, México; 7 Departamento de Patología Experimental, Instituto Nacional de Neurología y Neurocirugía “Dr. Manuel Velasco Suárez”, Ciudad de Mexico, 14269 Mexico; 8 Departamento de Neuroimagen, Instituto Nacional de Neurología y Neurocirugía “Dr. Manuel Velasco Suárez”, Ciudad de Mexico, 14269 Mexico

**Keywords:** Rathke cleft cyst, Pituitary adenoma, Xanthogranulomatous hypophysitis, Sellar collision lesions, Case report

## Abstract

The coexistence of three lesions in the sellar region is exceedingly rare. Only two cases with three histopathologically distinct lesions have been reported. However, here, we present a unique case of a 54-year-old female with pituitary adenoma (PA), xanthogranulomatous hypophysitis (XGH), and a Rathke cleft cyst (RCC). Clinically, the patient manifested symptoms of mass compression, such as moderate-intensity headaches and progressive visual acuity decrease. Relevant endocrinological evaluation revealed elevated free thyroxine levels without clinical manifestations. MRI revealed a suprasellar mass compatible with a macroadenoma. The patient underwent transsphenoidal endoscopic resection, resulting in a non-functional macroadenoma with associated XGH due to the rupture of RCC. Furthermore, in this article, we analyze the possible mechanisms involved in the pathogenesis of these lesions, emphasizing the type of spectrum to which they belong and the manifestations present.

**Supplementary Information:**

The online version contains supplementary material available at 10.1007/s10014-025-00504-4.

## Introduction

Sellar collision lesions, characterized by the coexistence of distinct histopathological entities in the sellar region, pose unique diagnostic and management challenges in clinical practice [1]. It has been suggested that these pathologies belong to a spectrum, manifesting at a specific time following the natural history of this group of disorders and may have a common embryological origin [2]. Here, we present a remarkable case involving a 54-year-old female patient with a large pituitary mass comprising a non-functional pituitary adenoma (PA), xanthogranulomatous hypophysitis (XGH), and a Rathke cleft cyst (RCC). This case presents a unique triple-sellar collision lesion, which the literature has not previously described. It prompts a detailed analysis of the potential pathogenetic mechanisms that could explain the simultaneous occurrence of these different lesions in the sellar region. Therefore, following the case presentation, we investigate the intricate interplay of factors that may contribute to developing this unique triple-sellar collision lesion, shedding light on the underlying pathophysiological mechanisms.

## Clinical summary

A 54-year-old woman was referred from a blindness clinic to our unit due to bitemporal hemianopsia. She reported a 4-year history of progressively decreasing visual acuity. In addition, she had experienced a gradual-onset headache for the past year, initially localized to the frontal region, which later became holocranial with a moderate intensity of 6/10. The headache improved with rest but worsened with stress and visual strain. Her medical history included a cyst resection in the posterior neck region performed 10 years before the current admission. A neuro-ophthalmological examination revealed mild bilateral optic atrophy secondary to compressive optic neuropathy, suggesting a chiasmal syndrome in the sellar region, likely of neoplastic origin. The remainder of the physical examination was unremarkable.

**Fig. 1:**
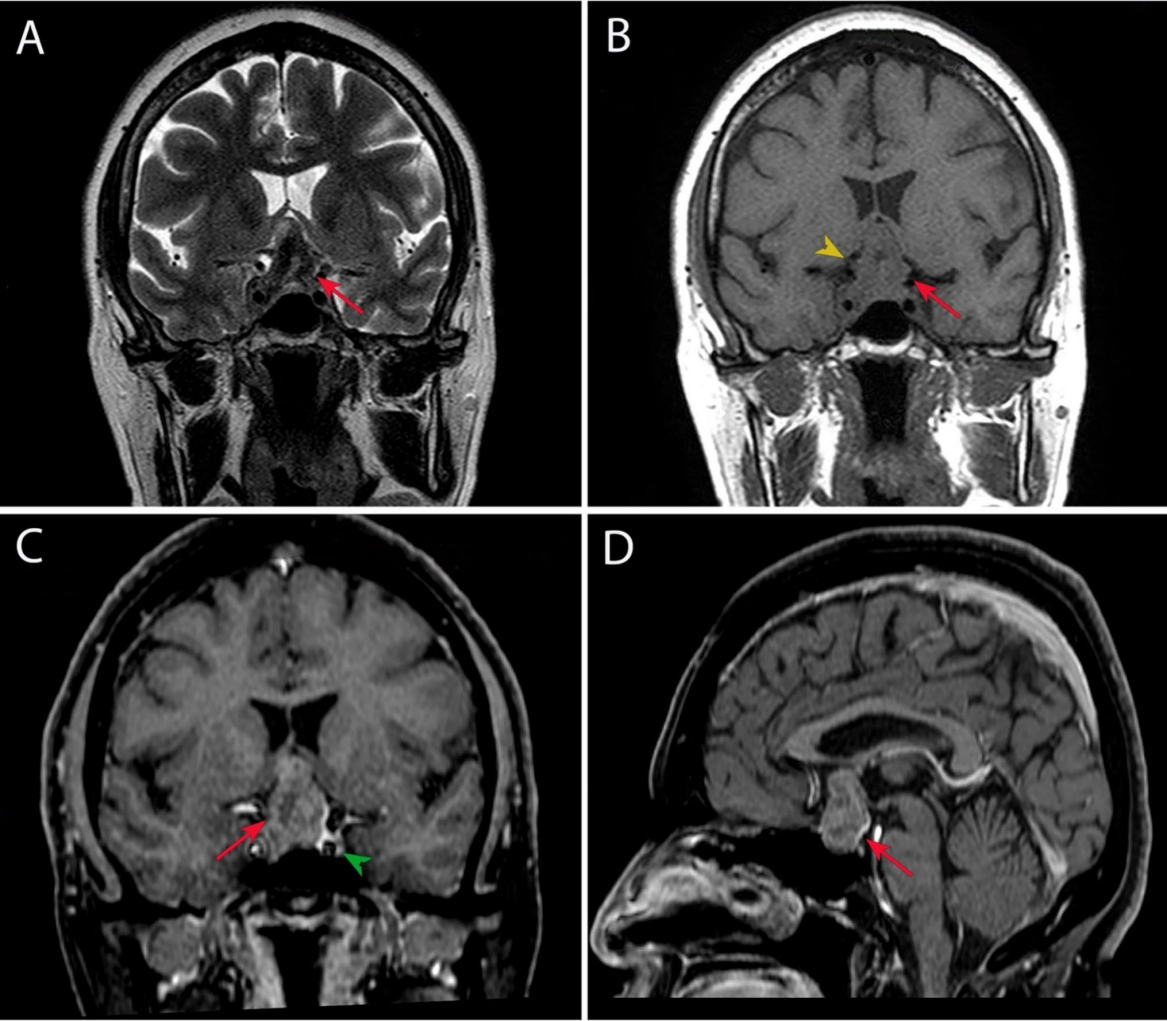
Radiological features of a heterogeneous space-occupying mass in different MRI sequences. **A** T2 coronal: heterogeneous space occupancy mass with hypointense areas in its periphery and isointense center (red arrow). **B** T1 simple coronal: an isointense space-occupying mass of irregular and undefined outline (red arrow) partially surrounds the right cavernous sinus with compression (yellow arrowhead) and dorsal displacement of the optic chiasm. **C** T1 coronal FAT SAT with contrast: moderate reinforcement is seen with central areas with necrosis (red arrow). The internal carotids are permeable (green arrowhead). **D** T1, sagittal with gadolinium: peripheral reinforcement is seen with central necrosis (red arrow); optic chiasm is not identified

The relevant endocrinological evaluation reported high free thyroxine levels (16.6 ng/dL), while the remainder of the hormonal profile was within normal levels including TSH (3.4 mUI/L), prolactin (11.2 ng/mL, undiluted), cortisol (7 µg/dL), IGF‑1 (79.12 ng/dL), and insulin (18.5 µU/mL), all in the absence of clinical manifestations. Magnetic resonance imaging (MRI) showed a 2.83 × 2.90 × 3.10 cm lesion in the suprasellar region compatible with a probable pituitary macroadenoma, that is, an occupying process with irregular contours, exhibiting hypointense areas in the periphery and isointense regions in the center, intensified by the contrast medium, as well as compression of the optic chiasm (Fig. 1). These findings led to a suspected diagnosis of a non-functional macroadenoma classified as Hardy–Vezina IIIC and KNOSP IIIA.

Two months after clinical examination, an endoscopic transsphenoidal resection was realized through an incision at the choana level and on the floor and ceiling of the nostril. A sphenoidotomy was performed to later mill the sellar floor and reach the dura, in which an opening was made. The lesion was identified as a macroadenoma; however, its gray appearance, yellow deposits, and diffuse calcifications also made it compatible with a craniopharyngioma. In addition, several irregular grayish-brown fragments of medium consistency inset with some soft regions of tissue measured 15 × 10 mm were found. The postoperative assessment demonstrated a 30–40% recovery of visual fields and a 60% improvement in visual acuity. MRI revealed a residual tumor adhered to the optic chiasm with invasion of both cavernous sinuses and a prior 50% resection. The hormonal profile persisted with an isolated elevation of free T4 (20.3 ng/dL). In contrast, TSH (3.44 mUI/L), LH (10.5 mUI/mL), FSH (23.1 mUI/mL), prolactin (3.8 ng/dL), cortisol (6.9 µg/dL), IGF-1 (118.4 ng/dL), GH (0.091 ng/dL), and insulin (18.30 µU/mL) remained within normal reference ranges.

## Pathological findings

**Fig. 2:**
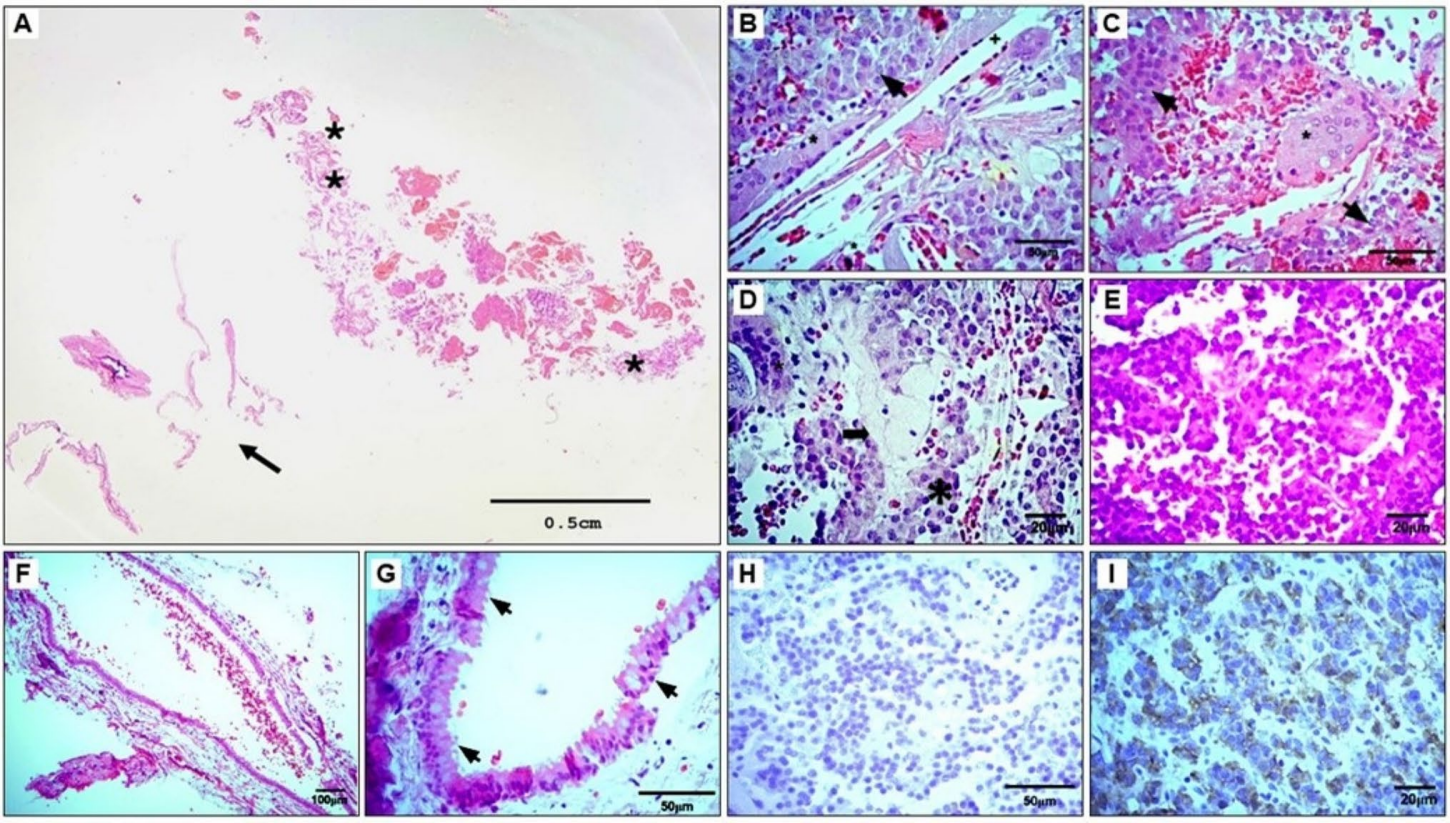
Histological findings of the triple sellar lesion. A Representative section of the complete biopsy of the lesion where remains of Rathke's pouch (arrow), presence of pituitary adenoma (*), and presence of areas of xanthogranulomatous inflammatory reaction (**) are observed. B Neoplastic cells characteristic of adenoma are (arrow) in direct relation to remains of cholesterol crystals ( +) and multinucleated giant cells (*); ×400. C Giant multinucleated cells (GMC, *) of foreign body type, with lipids in their vacuoles, are in direct relation with the neoplastic cells (arrow); ×400. D Adenoma cells are interspersed with foamy cells (arrow) and GMC (*); ×400. E Adenoma is composed of monotonous and non-typical large and eosinophilic cytoplasm cells ×400. F Rathke's pouch is observed, which is folded on itself, with a covering epithelium; ×100. G Respiratory epithelium is observed (pseudostratified ciliated columnar epithelium. Note the mucin-producing Goblet cells (arrows); ×400—hematoxylin and eosin (H&E) staining. H Representative photomicrograph against anti-ACTH, the rest of the antibodies against pituitary hormones did not demonstrate immunoreactivity (not shown); ×400. I Immunohistochemistry shows reactivity against epithelial membrane antigen (EMA); ×400

The pituitary adenoma exhibits a solid sheet-like pattern with homogeneous cells and nuclear uniformity. Some cells are arranged in perivascular pseudorosettes. There is no evidence of mitosis, pleomorphism, or atypia. The nuclei are round, centrally located, and occupy about two-thirds of the cell volume. They display finely granular chromatin, a thin nuclear membrane, and lack a visible nucleolus. The cytoplasm is eosinophilic with a well-defined membrane. Mild vascular ectasia is present. The tumor maintains a well-organized architecture, with uniform and intact cells. No necrosis, lymphocytic infiltration, or signs of pituitary apoplexy are observed. The hemorrhage seen is external to the tumor and corresponds to an extraction artifact, with no signs of tissue infarction (Fig. 2).

Adjacent to the adenoma, a Rathke’s cleft cyst is identified, lined with pseudostratified ciliated columnar respiratory epithelium containing goblet cells. No atypical or pleomorphic features are noted. The cyst contains fluid, and its peripheral vessels show congestion, suggesting a chronic inflammatory response to a foreign body, forming a xanthogranuloma. This xanthogranuloma alternates with areas of multinucleated giant cells within the pituitary adenoma. These giant cells have abundant cytoplasm and centrally located nuclei, some containing intracytoplasmic vacuoles. They make contact with adenomatous cells without phagocytosing them. In addition, giant cells with 10 to 15 or more nuclei are observed, some surrounding cholesterol crystals, and foamy macrophages containing lipid vacuoles (Fig. 2).

Immunohistochemistry revealed negative immunoreactivity of all hormones (growth hormone, GH; follicle-stimulating hormone, FSH; luteinizing hormone, LH; prolactin, PRL; adrenocorticotropic hormone, ACTH; thyroid-stimulating hormone, TSH), indicating a non-functional macroadenoma. Besides, the histological assays evidenced positive epithelial membrane antigen (EMA) of the RCC and macrophages of the xanthogranuloma positives for CD68. The primary antibodies used for immunohistochemistry were as follows: anti-ACTH antibody (1:100; Santa Cruz Biotechnology Inc., catalog number sc-57018), anti-PRL antibody (1:200; Santa Cruz Biotechnology, Inc., catalog number sc-20726), anti-GH antibody (1:200; Santa Cruz Biotechnology Inc., catalog number sc374266), anti-TSH antibody (1:200; Cell Marque, catalog number GMC21131020) anti-FSH beta antibody (1:100; Santa Cruz Biotechnology Inc., catalog number sc-374452), and anti-LH beta antibody (1:200; Santa Cruz Biotechnology, catalog number sc-373941).

## Discussion

The coexistence of three histopathologically distinct lesions in the sellar region is extremely rare. Most publications identify at least two pathologies, such as a pituitary adenoma with another lesion [1–6] or hypophysitis associated with the rupture of RCC [7–15]. Following PRISMA guidelines for systematic review, until Jan 25, 2024, we searched the medical literature reporting cases with more than two sellar lesions written in English or the Spanish language using the PubMed electronic database and the keywords "Sellar lesions OR Sellar tumor lesions" AND "Rathke's cleft Cyst OR craniopharyngioma neoplasm" AND "pituitary adenoma OR pituitary neoplasm" OR "Xanthogranulomatous Hypophysitis OR Hypophysitis." We also performed manual searches of additional relevant studies in Scopus, identifying only two reported cases of three histopathologically distinct lesions (Table 1, Online Resource 1) in the Sellar region; both cases were also reported in female patients aged 71 and 35, respectively. The first of them reported the presence of a non-functioning adenoma accompanied by the presence of lymphocytic hypophysitis and primary pituitary lymphoma. In contrast, the second reported a growth hormone-producing microadenoma accompanied by the presence of lymphocytic hypophysitis and RCC [16, 17]. However, here, we describe a case that presents this for the first time: PA, RCC, and the rare XGH concurrently.

**Table 1:** Triple sellar collision lesions that are reported in the literature

Country (Year)	Gender/ Age	MRI	Surgery approach	Microscopic findings and Diagnosis (Dx)	Postoperative outcomes	Proposed neuropathological mechanisms
USA (2011) [17]	Female/ 71 y/o	The suprasellar mass measured 2.3 × 2.2 × 1.7 cm and compressed the optic chiasm	Transsphenoidal resection	The examination showed loose cohesive cells with numerous mitotic figures associated with lymphocytic hypophysitis adjacent to the tumor. The tumor was positive for B cell markers (CD20 and CD79 alpha). The positive Ki67 marker suggests active proliferation in the tumoral area, while the positive Bcl6 marker confirms the origin of malignant lymphocytes in the germinal center. Moreover, a non-functional adenomatous area near the lymphoma was recognized Dx. Non-functional adenoma + lymphocytic hypophysitis + primary pituitary lymphoma	The patient received radiotherapy, and after 1 year, she was completely asymptomatic	The lymphoma might originate from the CNS, the lymphoid tissue, or normal lymphocytes may migrate to the sellar region due to inflammatory processes of lymphocytic hypophysitis and trigger malignant transformation. The non-functional adenoma may be the source of elevated GH, IGF-I, IGF-II, and insulin receptors in normal lymphocytes, potentially contributing to this malignant transformation
USA (2017) [16]	Female/ 35 y/o	Suprasellar mass measured 2.1 × 1.9 × 2.7 cm	Transsphenoidal resection	The histopathological analysis shows a microadenoma positive for GH, an RCC with ciliated epithelium, and lymphocytic hypophysitis identified by the proliferation of inflammatory cells marked with the common leukocyte antigen (CD45) Dx. Growth hormone-producing microadenoma + lymphocytic hypophysitis + RCC	Recovery was complicated by right thalamic stroke, subarachnoid hemorrhage, transient diabetes insipidus, and persistent CSF leak requiring multiple repairs. Postoperatively, the patient required glucocorticoid, thyroid hormone, and estrogen replacement therapy	The rupture of a RCC could trigger an inflammatory reaction, potentially leading to pituitary cell dysfunction and subsequent hyperplasia or adenoma formation
Mexico (Present case)	Female/ 54 y/o	2 cm diameter suprasellar mass compatible with a macroadenoma	Endoscopic transsphenoidal resection	PA not reactive to pituitary hormone markers, accompanied by acute and chronic inflammatory response, presence of remnants a RCC wall (EMA +), as well as cholesterol clefts and foreign-type multinucleated giant cells with plenty of foamy cells (CD68 +) indicative of xanthogranuloma Dx. Nonfunctional macroadenoma + RCC + XGH	The patient presented partial improvement in symptoms; no other complications were reported	RCC originates from alterations in the embryonic development of the pituitary gland, making it more susceptible to chronic inflammation. This inflammation can lead to the formation of a PA and the weakening of the cyst wall, making it more prone to rupture and the subsequent release of its contents, potentially causing XGH

*Dx* Diagnosis, *CNS* Central Nervous System, *GH* growth hormone, *IGF-I *Insulin-like growth factor type I, *IGF-II* Insulin-like growth factor type II, *PA* Pituitary adenoma , *RCC *Rathke cleft cyst, *CSF* cerebrospinal fluid , *XGH* xanthogranulomatous hypophysitis

The pituitary gland is the primary endocrine organ regulating the body's hormonal balance. Its embryonic development involves the intricate interaction of different tissues. The anterior pituitary originates from the oral ectoderm (Rathke's pouch), while the posterior pituitary develops from the neural ectoderm of the diencephalon during weeks 3–4 of fetal development [18]. It is located in the hypophyseal fossa (*sella turcica*), a mid-skull region with great structural complexity due to the presence of surrounding structures, such as the internal carotid artery, cavernous sinus, optic chiasm, hypothalamus, and the pituitary gland [19, 20]. Sellar lesions are any neoplastic or tumor-like mass originating in any of the components that make up this region [20]. They represent frequent lesions, an incidental finding in up to 10–30% of MRI scans indicated for another cause [21, 22], and a finding in 10% of autopsies [23].

In most cases, they usually present as single lesions; however, in exceptional conditions (< 2% of cases), they present concomitantly as a collision tumor [5]. Clinical manifestations are nonspecific and are usually secondary to mass effects on adjacent anatomical structures or hormonal abnormalities [24]. The primary differential diagnoses for suprasellar lesions include pituitary adenomas, Rathke’s cleft cysts, craniopharyngiomas, lymphocytic hypophysitis, metastatic tumors, and less common entities, such as granular cell tumors or abscesses. Given the limited specificity of imaging studies, an accurate diagnosis and appropriate management require a comprehensive evaluation that integrates clinical, biochemical, and histopathological findings [25, 26].

### Pituitary adenomas

PAs are the most common sellar lesion, accounting for up to 90% of cases in adults and representing 10–15% of all brain tumors [25, 26]; they are benign clonal neoplasms of the neuroendocrine cells of the adenohypophysis [27]. They are classically classified as micro (< 10 mm diameter) and macroadenomas (> 10 mm diameter), as well as hormone-active (functional) and hormone-inactive (non-functional) adenomas depending on whether they are associated with hormone production [20, 28], 70% of them being hormonally active [29]. On MRI, the presence of a homogeneous isointense image on T2 and T1 is typical [30], which contrasts with what was observed in our case, where a heterogeneous image was presented, possibly explained by the size of the lesion. However, when performing a contrast-enhanced MRI, enhancement of the lesion was observed, constituting a gold standard for diagnosing PA [29].

Histologically, these lesions are characterized by rounded epithelioid cells with granular cytoplasm, round nuclei with finely dispersed chromatin, and multiple distinct nucleoli [27]. The above corresponds to what was found in our patient's mass, where we observed foci of the lesion characterized by acute and chronic inflammatory responses, pseudostratified ciliated columnar epithelium, foamy macrophages, cholesterol clefts, and dystrophic calcifications, along with non-functional pituitary adenoma with no hormone activity. (Fig. 2D). In our case, despite finding increased free thyroxine in the endocrinological profile, we did not find immunoreactivity to any of the pituitary hormones, and given that the thyroid-stimulating hormone values were found within normal ranges, the most probable cause for this endocrinological finding would be an episode of asymptomatic thyroiditis [31]. According to the World Health Organization (WHO), adenoma classification requires identifying transcription factors [28]. Therefore, and as a limitation of our case, we cannot fully establish a null cell adenoma since 1/5 of clinically non-functioning adenomas are negative for all anterior pituitary hormones but positive for the steroidogenic factor-1 (SF-1), being indicative of a gonadotroph lineage [32, 33].

### Rathke cleft cyst

RCCs are non-neoplastic cystic lesions arising from remnants of Rathke's pouch, formed between the anterior and posterior lobes of the pituitary gland. Symptomatic cases are rare, and their epidemiology is uncertain [34]. Macroscopically, the cyst is encapsulated in a delicate membrane, and the contents can be diverse, with 65% having a proteinaceous content formed by hemosiderin, cholesterol, mucin, and other proteins [35]. The content of the cyst is of interest, as variations have been observed in the clinical and imaging findings, cysts with proteinaceous content being the ones that are associated with the presence of symptoms such as headache [36, 37], which coincides with our case, where the presence of a proteinaceous content associated with symptomatology was present. The MRI signal depends on the cyst content; on T1-weighted images, RCCs may present both hypo- or hyperintense; on T2-weighted sequences, about 70% appear hyperintense, while 30% are iso-to-hypointense. These are characteristics of an intracystic non-enhancing nodule and enhanced rim of compressed pituitary surrounding the cyst after contrast administration [20, 34]. In the present case, the imaging findings do not comply with the above; however, it should be noted that in the presence of a collision tumor concomitant to a PA, it is difficult to establish the differential diagnosis by imaging [2, 5]. In fact, it was observed that of a total of 39 cases, only 15 presented two different magnetic MRI signals that allowed to suspect both lesions [6].

Histologically, the cyst's wall is formed by a simple or pseudostratified cuboidal or columnar epithelium, which expresses EMA, a transmembrane glycoprotein typically expressed in luminal epithelial cells that provides barrier functions. The epithelium of the cyst also contains ciliated cells and goblet cells, the last responsible for the production of the mucinous material that accumulates inside the cyst [34]. In the present case, we found remains of the cyst's wall, which is most likely due to a spontaneous cyst rupture. It has been observed that the cyst wall undergoes cyclical changes of inflammation that cause bleeding and subsequent rupture and degeneration of the cyst and can even trigger xanthogranulomatous changes [12, 13, 38]. The above indicates that RCC, xanthogranulomas, and craniopharyngiomas constitute a spectrum of the same entity [39]. Nonetheless, rupture of the cyst could be favored by other mechanisms, including increased intra-cystic pressure due to dysregulation of content secretion–reabsorption, decreased cyst wall thickness related to chronic inflammatory changes, or mass effect exerted by pressure with other structures [9, 40, 41].

### Hypophysitis

Hypophysitis is the acute or chronic inflammation of the pituitary gland and is classified as primary (idiopathic) or secondary to *sella turcica* and parasellar region lesions, systemic diseases, or drugs [25]. It is a rare disease compared to other pituitary lesions, with an estimated prevalence of 0.2–0.88% [42]. Histologically, it can be classified as lymphocytic (the most common form), granulomatous, xanthomatous, IgG-4 related, necrotized, and mixed forms such as XGH (the least frequent) [42, 43]. In MRI studies, stalk thickening and homogenous enlargement of the pituitary gland are typically observed; however, imaging is not specific [43].

The granulomatous form is typically idiopathic or secondary to systemic diseases, such as tuberculosis or Langerhans disease [43]. Histologically, it is characterized by numerous multinucleated giant cells, calcium deposits, lymphocytes, and histiocytes with granuloma formation [11]. The above is characteristic of a response to a foreign body [44]. On the other hand, the xanthomatous variety is frequently secondary to local processes, commonly the rupture of an RCC [13]. Histologically, it presents CD68-positive foamy cells, cholesterol clefts, and hemosiderin deposits [45]. Here, we report a case of an extremely rare subtype of hypophysitis called XGH, exhibiting xanthomatous and granulomatous features characterized by the presence of cholesterol clefts, lymphocytes, plasma cells, hemosiderin deposits, fibrosis, foreign body giant cells, eosinophilic remnants, caseating and necrotizing granuloma, accumulation of foamy macrophages, and granuloma formation [46, 47]. This variety of hypophysitis may be primary with an autoimmune etiology or secondary as a reactive degenerative response to an epithelial lesion or as a part of a multiorgan systemic [46]. This case is particularly notable, because it presents alongside a PA and an RCC, suggesting a complex network of pathophysiological mechanisms. The concurrent development of these three lesions may indicate a shared inflammatory or immune response within the hypophyseal fossa, possibly driven by the local microenvironment or a systemic predisposition to multiple pathologies. Understanding the histopathological interactions and triggers leading to such unusual presentations could provide deeper insights into pituitary disease mechanisms and guide more effective diagnostic and therapeutic approaches.

### Explaining the coexistence of a collision tumor

Multiple theories have been proposed to explain the existence of collision tumors in the sellar region [1, 5], particularly the coexistence of PA with RCC is the most frequent finding. Furthermore, prolactinomas or somatotroph lineage adenomas are more common than non-functioning adenomas, such as the one presented in this case [1]. However, none of those theories explain the presence of all collision tumors, so their existence as a coincidence remains feasible. Therefore, our objective is to discuss the interrelationship between these lesions rather than explaining their same origin.

**Fig. 3:**
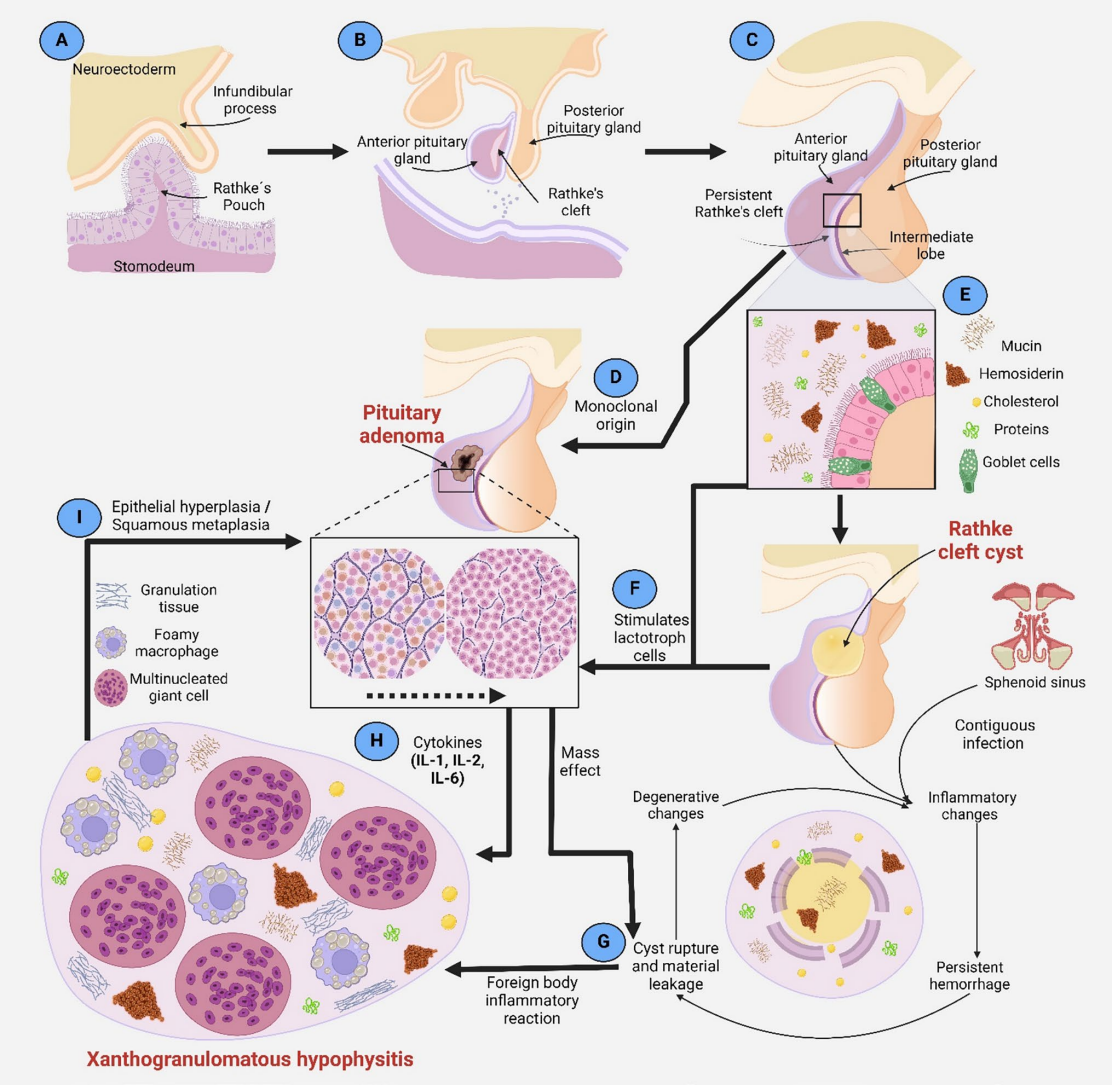
Proposed pathological mechanisms of the possible relationship between pituitary adenoma, Rathke cleft cyst, and xanthogranulomatous hypophysitis. Development of the pituitary gland at (**A**) 4 and (**B**) 8 gestation weeks, as well as in (**C**) the adult stage. **D, F, I** Possible origins of the pituitary adenoma. **E** Development of the Rathke cleft cyst after the accumulation of the secretion of cellular remnants in the Rathke's cleft. **G, H** Possible origins of xanthogranulomatous hypophysitis. This figure was created with BioRender.com

The development of the pituitary gland is a complicated process that involves the function of various genes and signaling pathways in the first 4–8 weeks of gestation (Fig. 3A, B) [27], during which, occur the formation of the two main lobes of the gland and closure of Rathke's cleft in the *pars intermedia*. The cause of the incomplete closure of this cleft, which would give rise to the formation of RCC due to the accumulation of material secreted by its wall, has not yet been identified (Fig. 3C, E)[2, 40]. Specifically, no mutations in the genes or signaling pathways involved in the development of the pituitary gland have been related to this phenomenon or the development of PAs. Interestingly, in addition to PAs with a familial predisposition, it has been observed that mutations in the *GNAS* and *USP-8* genes are the only significant molecular mechanisms in sporadic somatotroph and corticotroph PAs, probably by favoring the mitogenic signal or by deregulation of the cell cycle, respectively [27, 48].

Complementary studies in animal models have identified that mutations in the *Prop-1* gene are associated with a higher incidence of PAs or RCC independently and without increasing the frequency of a collision tumor [49]. However, we can suggest that there is an interaction of RCC content that could stimulate the lactotrophic cells of the adenohypophysis and cause the development of an adenoma (Fig. 3F) [50].

On the other hand, cysts with mucinous content tend to superinfection [35] and consequent inflammation and rupture, which would cause a constant leak of cyst material (Fig. 3G). The continuous exposure of the pituitary epithelium to the cyst's contents would cause an inflammatory response [7, 9], in the present case of the foreign body type, mediated by mucin and hemosiderin [14, 39]. Furthermore, the presence of a PA could stimulate or trigger this inflammatory response, since it has been observed that PAs secrete pro-inflammatory cytokines (IL-1, IL-2, and IL-6) associated with the development of hypophysitis (Fig. 3H)[51] or even the inflammation could be secondary to silent apoplexy of the PA [39]. Finally, this chronically maintained inflammatory response could cause epithelial hyperplasia and squamous metaplasia of the pituitary gland and subsequent development or growth of a PA (Fig. 3I) [16]. In this case, the coexistence of a PA, RCC, and XGH highlights a complex interplay of pathological mechanisms. The RCC may have initiated inflammation that led to PA development, and pro-inflammatory cytokines from the PA could have exacerbated this inflammation, facilitating XGH formation.

In addition, the continuous exposure of pituitary tissue to the leaking RCC content could have perpetuated the inflammatory response. This sequence of events implies that each lesion influences the development and evolution of the others. Understanding these dynamics is crucial for improving the diagnosis and treatment of complex pituitary lesions.

## Supplementary Information

Below is the link to the electronic supplementary material.

Supplementary file1 (DOCX 42 KB)

## Data Availability

No datasets were generated or analyzed during the current study.
