# Supplementary figures and images for: Triple sellar collision lesion: a unique case of pituitary adenoma, Rathke cleft cyst, and xanthogranuloma—case report and systematic review of the literature
Source: Brain Tumor Pathol. 2025 Jun 6;42(4):143–52. doi: 10.1007/s10014-025-00504-4 (PMC12518419; doi:10.1007/s10014-025-00504-4)

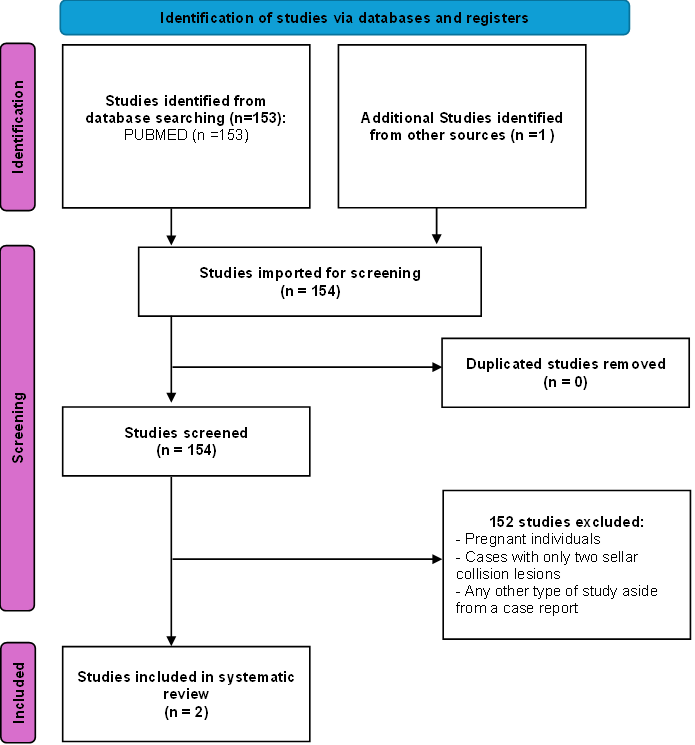


**Online resource 1.** Algorithm for database search and article selection

Supplement: Supplementary file 1 — Supplementary file1 (DOCX 42 KB) [file 10014_2025_504_MOESM1_ESM.docx]
